# Mass spectrometry-based identification and whole-genome characterisation of the first pteropine orthoreovirus isolated from monkey faeces in Thailand

**DOI:** 10.1186/s12866-018-1302-9

**Published:** 2018-10-17

**Authors:** Nathamon Kosoltanapiwat, Onrapak Reamtong, Tamaki Okabayashi, Sumate Ampawong, Amporn Rungruengkitkun, Tipparat Thiangtrongjit, Narin Thippornchai, Pornsawan Leaungwutiwong, Aongart Mahittikorn, Hirotake Mori, Thanada Yoohanngoa, Prechaya Yamwong

**Affiliations:** 1 Department of Microbiology and Immunology, Faculty of Tropical Medicine, Mahidol University, 420/6 Ratchawithi Road, Ratchathewi, Bangkok, 10400 Thailand; 2 Department of Molecular Tropical Medicine and Genetics, Faculty of Tropical Medicine, Mahidol University, 420/6 Ratchawithi Road, Ratchathewi, Bangkok, 10400 Thailand; 3 Department of Veterinary Sciences, Faculty of Agriculture, University of Miyazaki, Gakuen-kibanadai-nishi-1-1, Miyazaki, 889-2192 Japan; 4 Mahidol-Osaka Center for Infectious Diseases (MOCID), Research Institute for Microbial Diseases, Osaka University, 3-1 Yamadaoka, Suita, Osaka, 565-0871 Japan; 5 Department of Tropical Pathology, Faculty of Tropical Medicine, Mahidol University, 420/6 Ratchawithi Road, Ratchathewi, Bangkok, 10400 Thailand; 6 Department of Protozoology, Faculty of Tropical Medicine, Mahidol University, 420/6 Ratchawithi Road, Ratchathewi, Bangkok, 10400 Thailand

**Keywords:** Monkey, Macaque, *Macaca fascicularis*, Orthoreovirus, Hepatitis E virus, Mass spectrometry

## Abstract

**Background:**

The pteropine orthoreovirus (PRV) was isolated from monkey (*Macaca fascicularis*) faecal samples collected from human-inhabited areas in Lopburi Province, Thailand. These samples were initially obtained to survey for the presence of hepatitis E virus (HEV).

**Results:**

Two virus isolates were retrieved by virus culture of 55 monkey faecal samples. Liquid chromatography-tandem mass spectrometry (LC-MS/MS) was successfully used to identify the viruses as the segmented dsRNA orthoreovirus. Phylogenetic analysis of the Lopburi orthoreovirus whole-genomes revealed relationships with the well-characterised PRVs Pulau (segment L1), Cangyuan (segments L2, M3 and S3), Melaka (segments L3 and M2), Kampar (segments M1 and S2) and Sikamat (segments S1 and S4) of Southeast Asia and China with nucleotide sequence identities of 93.5–98.9%. RT-PCR showed that PRV was detected in 10.9% (6/55) and HEV was detected in 25.5% (14/55) of the monkey faecal samples.

**Conclusions:**

PRV was isolated from monkey faeces for the first time in Thailand via viral culture and LC-MS/MS. The genetic diversity of the virus genome segments suggested a re-assortment within the PRV species group. The overall findings emphasise that monkey faeces can be sources of zoonotic viruses, including PRV and HEV, and suggest the need for active virus surveillance in areas of human and monkey co-habitation to prevent and control emerging zoonotic diseases in the future.

**Electronic supplementary material:**

The online version of this article (10.1186/s12866-018-1302-9) contains supplementary material, which is available to authorized users.

## Background

In some areas in Lopburi Province, Thailand, humans and monkeys, mostly macaques, live in close contact. In these areas, faeces excreted by the animals can unavoidably contaminate the human environment. Faeces are sources of pathogens, including bacteria, parasites and viruses. Most enteric viruses typically present in faeces are non-enveloped viruses that can exist outside the host for several days [1, 2]. Some of these are zoonotic viruses that can infect both humans and animals. Hence, in areas in which humans and animals co-inhabit, the potential for zoonotic transmission is elevated, requiring observation and control.

Pteropine orthoreovirus (PRV) is a member of the genus *Orthoreovirus* and family *Reoviridae*. *Reoviridae* is a large family of non-enveloped, icosahedral, segmented dsRNA viruses that infect a wide range of hosts, i.e. fungi, plants, insects, molluscs, fish, reptiles, birds and mammals, including humans [3]. *Orthoreovirus*, which contains 10 genome segments (three large, three medium and four small segments), consists of five species: mammalian orthoreovirus (MRV), avian orthoreovirus (ARV), PRV [formerly known as Nelson Bay orthoreovirus (NBV)], baboon orthoreovirus and reptilian orthoreovirus [4, 5]. Of these, MRV is the only species that does not exert a syncytial cytopathic effect (CPE) in cell culture; therefore, it is called a non-fusogenic orthoreovirus. MRV is the prototypic orthoreovirus that causes diseases in mammals, including humans and monkeys [3]. The first isolation of PRV occurred in 1970 from a grey-headed flying fox (*Pteropus poliocephalus*) in Nelson Bay, Australia [6]. Its characteristics are intermediate between MRV and ARV, in which it is a fusogenic orthoreovirus that infects mammals but does not kill chicken embryos [3]. It is therefore called a mammalian fusogenic orthoreovirus. Later, other PRVs were isolated from bats and humans. Pulau virus, the second member of the NBV species group, was isolated in 1999 from pooled urine samples of the fruit bat *Pteropus hypomelanus* in Tioman Island, Malaysia [7]. Bat-associated Melaka and Kampar orthoreoviruses with genome sequences related to NBV were isolated in 2006 in Malaysia from patients with acute respiratory disease. The reports of these two viruses suggested the ability of PRV to cause disease in humans and evidenced its human-to-human transmission potential [8, 9]. Subsequently, new strains of PRV were consecutively isolated from humans and bats, including patients with respiratory tract infection from Hong Kong [10, 11], Japan [12] and Malaysia [5] and bats from China [13, 14], Italy [15] and, most recently, the Philippines [16]. It must be noted that the infected patients from Hong Kong and Japan had histories of travel to Indonesia, and the PRV-positive bats in Italy were also imported from Indonesia. Thus far, excluding NBV, which originated from Australia, PRV has only been isolated in Southeast Asia (Malaysia, Indonesia and the Philippines) and China.

Meanwhile, mass spectrometric techniques have been increasingly utilised in virus studies. Mass spectrometry (MS), peptide mass fingerprinting and protein profiling via time-of-flight mass spectrometry (TOF MS) have been applied in studies of both human and plant viruses such as poliovirus, rhinovirus, tobacco mosaic virus and brome mosaic virus [17–19]. However, the use of peptide mass fingerprinting to identify unknown viruses is restricted by limitations of viral peptide mass fingerprint databases and the capability of TOF MS to identify small amounts of viral peptides when disturbed by mammalian proteins from cell culture. To overcome these limitations, protein separation techniques such as gel electrophoresis and liquid chromatography (LC) in combination with MS are applied. MS, in particular tandem MS (MS/MS), is a sensitive method for analysing protein mixtures [20]. Two-dimensional (2D) electrophoresis in combination with high-performance liquid chromatography-tandem mass spectrometry (HPLC-MS/MS) was used to identify an uncharacterised virus in an experimentally infected tobacco plant protein extract [21].

The initial aim of this study was to survey for the presence of hepatitis E virus (HEV) in monkey faeces. HEV is a food- and water-borne, non-enveloped, positive-sense RNA virus of the *Hepeviridae* family. The virus causes diseases ranging from acute self-limiting illnesses to fulminant hepatitis in humans [22]. In Thailand, sporadic cases of HEV infection have been reported. Nationwide HEV sero-surveillance revealed an HEV seroprevalence rate of 3–26% in Thai provinces [23]. Focussing on Lopburi Province, 37% of people in a surveyed population were anti-HEV IgG positive, with positivity associated with pork consumption and the presence of swine farms in the area [24]. HEV is the only hepatitis virus that exhibits a zoonotic potential, with pigs as a primary reservoir [22]. In addition, HEV has been detected in wild boars, wild deer, mongooses, rabbits, rats and goats in various countries in Asia and Europe [22, 25, 26]. Non-human primates (NHPs) have been demonstrated to be experimentally infected by HEV and to secrete the virus. Although it has been suggested that monkeys serve as an asymptomatic reservoir of HEV [27], the prevalence of HEV in wild monkeys in Thailand has not yet been elucidated.

In this study, we report the first isolation and characterisation of an orthoreovirus from monkey faeces collected from areas occupied by humans in Lopburi Province, Thailand. Two virus isolates were retrieved via viral culture. These isolates named Lopburi01 and Lopburi02 were identified using SDS-PAGE, LC-MS/MS and protein database searches, and their genomes were characterised by phylogenetic analysis. In addition, the detection of PRV and HEV by RT-PCR in all monkey samples was reported.

## Results

### Isolation of Lopburi01 and Lopburi02 viruses from monkey faeces

**Fig. 1:**
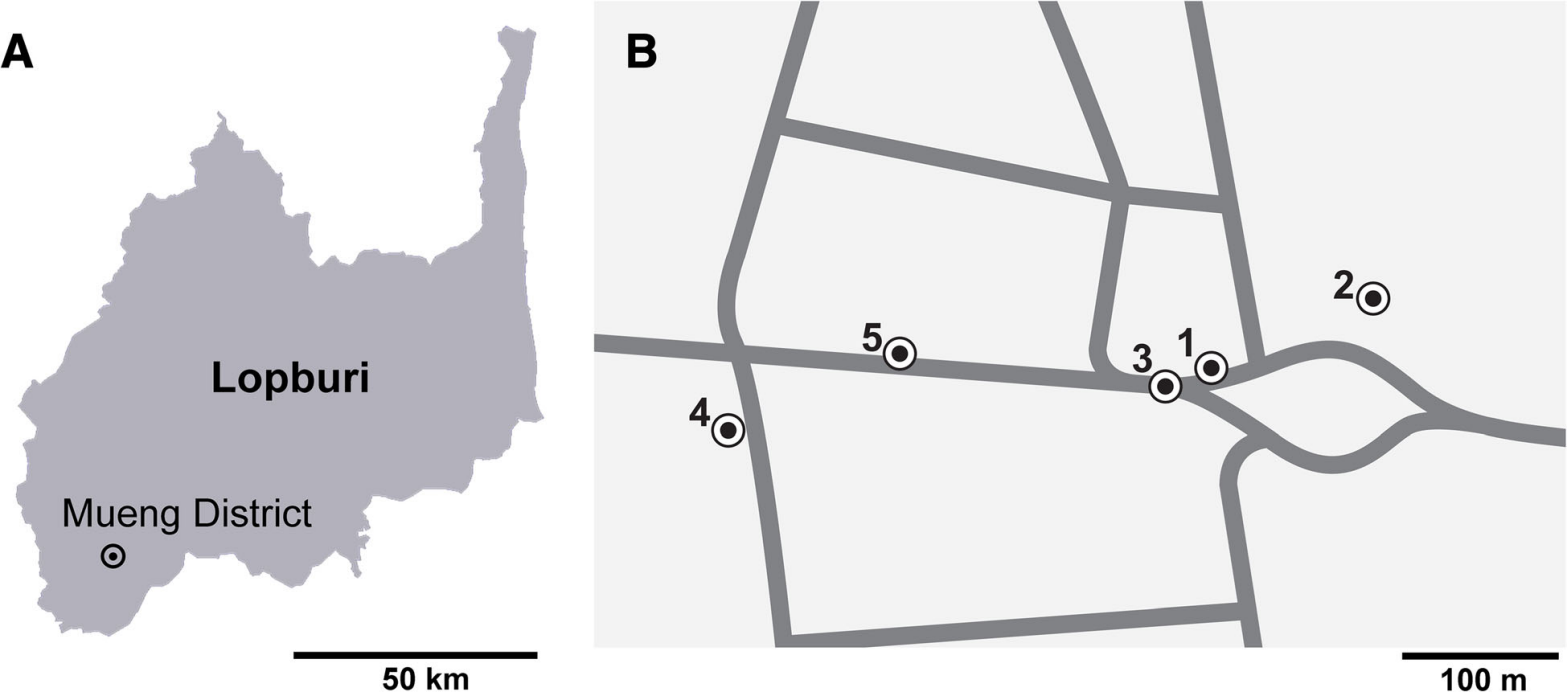
Faecal sample collection sites. **a** Circle indicates Mueng District in the Lopburi Province, Thailand. **b** Circles indicate locations of sample collection sites in Mueng District

**Fig. 2:**
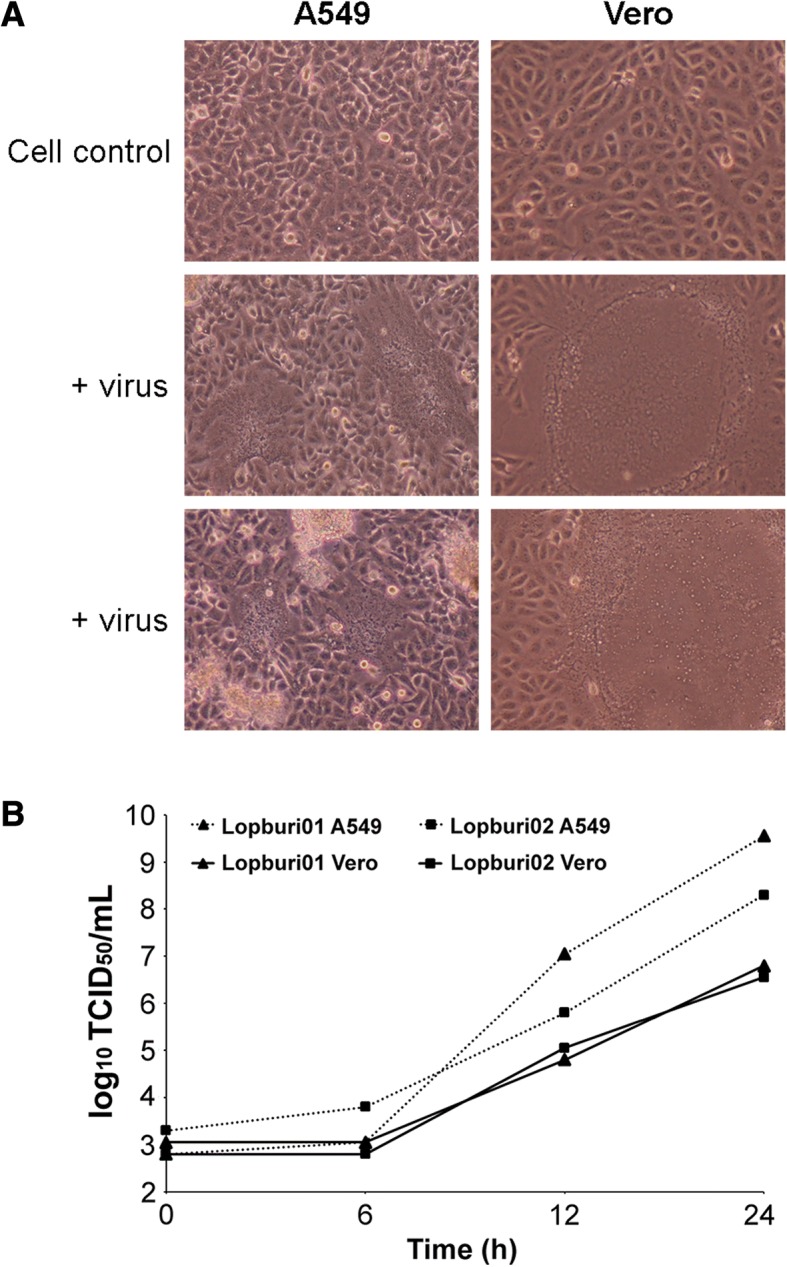
Cytopathic effect (CPE) and growth kinetics of the Lopburi viruses in A549 and Vero cells. **a** The cells were inoculated with the virus (Lopburi02) for 24 h. A syncytial CPE was observed in both infected Vero and A549 cell monolayers but not in uninfected cells. Similar data were obtained for the Lopburi01 isolate. **b** A549 and Vero cells were infected with the Lopburi01 and Lopburi02 viruses at MOI of 0.5 for 24 h. Viral growth curves were obtained by titration of the virus culture supernatants collected at 0, 6, 12, and 24 h. The result is a representative of 2 independent experiments

Of 55 monkey faecal samples, 2 virus isolates were retrieved by virus culture in A549 cells. After virus culturing for 3 days, CPEs were observed in samples collected from sites 1 (temple area) and 5 (working area) (Fig. 1 and Table 1). Culture supernatants were harvested and sub-passaged in A549 and Vero cells. CPEs observed in both cell types infected with the two isolates exhibited the same characteristic, syncytium formation (Fig. 2a). The virus isolates were named Lopburi01 and Lopburi02, respectively. Growth kinetics of the viruses in A549 and Vero cells were investigated (Fig. 2b). The Lopburi viruses showed a higher degree of replication in A549 cells than in Vero cells. RNA extracted from the culture supernatants of Lopburi01 and Lopburi02 were tested for HEV, herpesvirus, enterovirus, rotavirus, enteric adenovirus, norovirus and astrovirus, but negative results were obtained.

**Table 1:** Details of the monkey faecal sample collection, virus isolation and virus detections

Sample collection				Nested RT-PCR	
Site	Place	Number	Virus isolation	PRV positive	HEV positive
1	Temple area	13	1	1 (7.7%)	3 (23.1%)
2	School area	4	0	1 (25%)	1 (25%)
3	Temple area	14	0	3 (21.4%)	6 (42.9%)
4	House area	12	0	0	1 (8.3%)
5	Working area	12	1	1 (8.3%)	3 (25%)
	Total	55	2	6 (10.9%)	14 (25.5%)

### Physicochemical and morphological properties of the isolated viruses

**Fig. 3:**
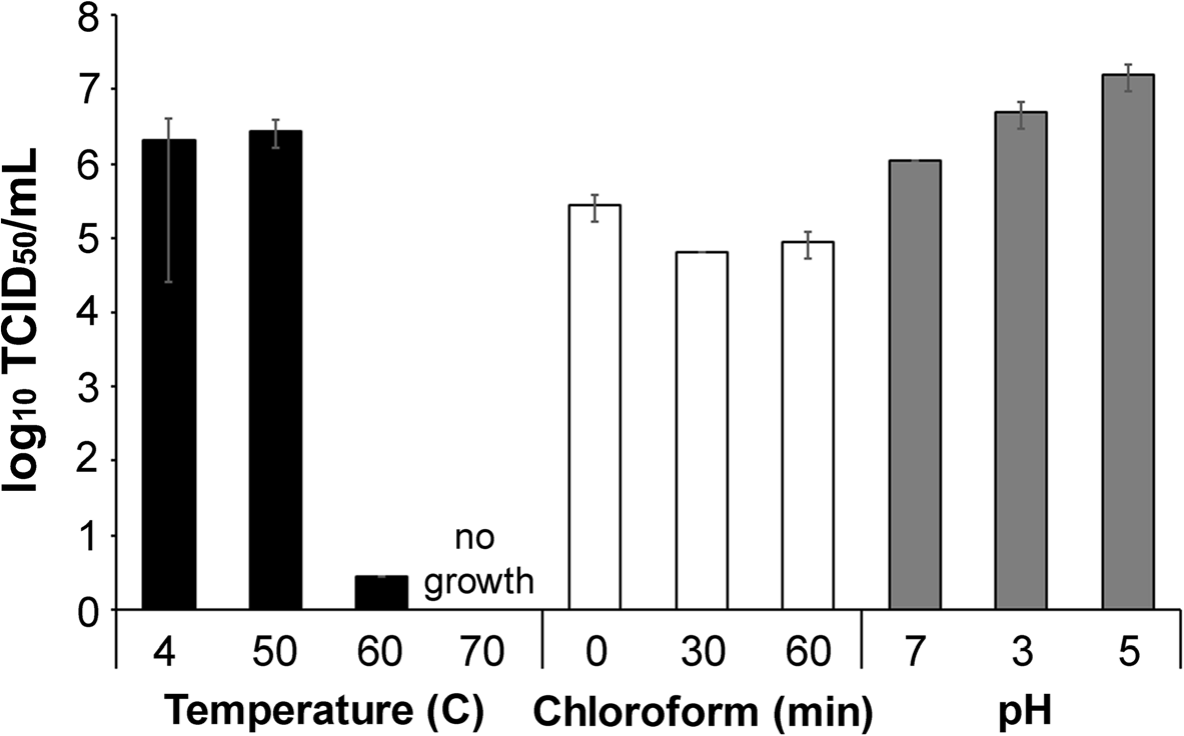
Physicochemical properties of the Lopburi virus. Culture supernatants of the Lopburi02 virus were exposed to different temperatures for 1 h (black bars), chloroform for 30 or 60 min (white bars) and different pH conditions for 20 h (grey bars). After treatment, the viruses were titrated in Vero cells, and their levels were quantified as TCID_50_/mL. Two independent experiments were performed for each assay. Similar findings were observed for the Lopburi01 isolate

To further characterise the Lopburi01 and Lopburi02 viruses, their stabilities under temperature, chloroform and acid stress were compared with those of untreated controls. As shown in Fig. 3, the Lopburi02 virus resisted exposure to temperatures up to 50 °C, whereas it was destroyed by exposure to temperatures exceeding 60 °C for 1 h. Treatment with chloroform for 30 and 60 min did not inactivate the virus, although a slight decrease in TCID_50_ was observed compared with that of the untreated virus (0 min). The virus was not inactivated by exposure to pH 3 or pH 5 for 20 h. Moreover, an approximately 1 log_10_ increase in TCID_50_/mL was observed when the virus was exposed to acidic treatment (pH 3 and pH 5) compared with the effects of PBS (pH 7). The same results were observed with the Lopburi01 isolate. Taken together, the results suggested that Lopburi01 and Lopburi02 are non-enveloped viruses that resist chloroform and acidic pH.

**Fig. 4:**
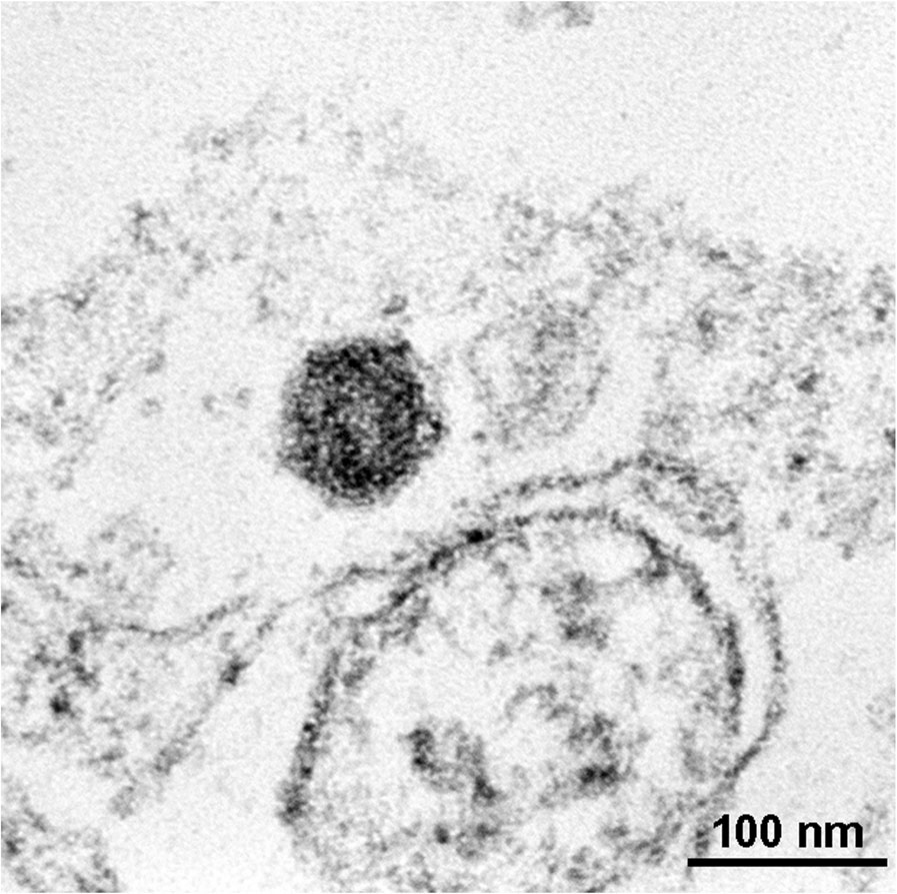
Transmission electron micrograph of the Lopburi virus. The Lopburi02 virus was cultured in Vero cells. A viral particle characterised by an icosahedral structure was observed in the cytoplasm of the infected cell pellet

Transmission electron microscopy was used to determine the morphology of Lopburi02 in Vero cell pellets. Non-enveloped virus particles of 50–80 nm in size with an icosahedral structure were observed in the Vero cell cytoplasm (Fig. 4).

### Identification of the Lopburi virus by LC-MS/MS

**Fig. 5:**
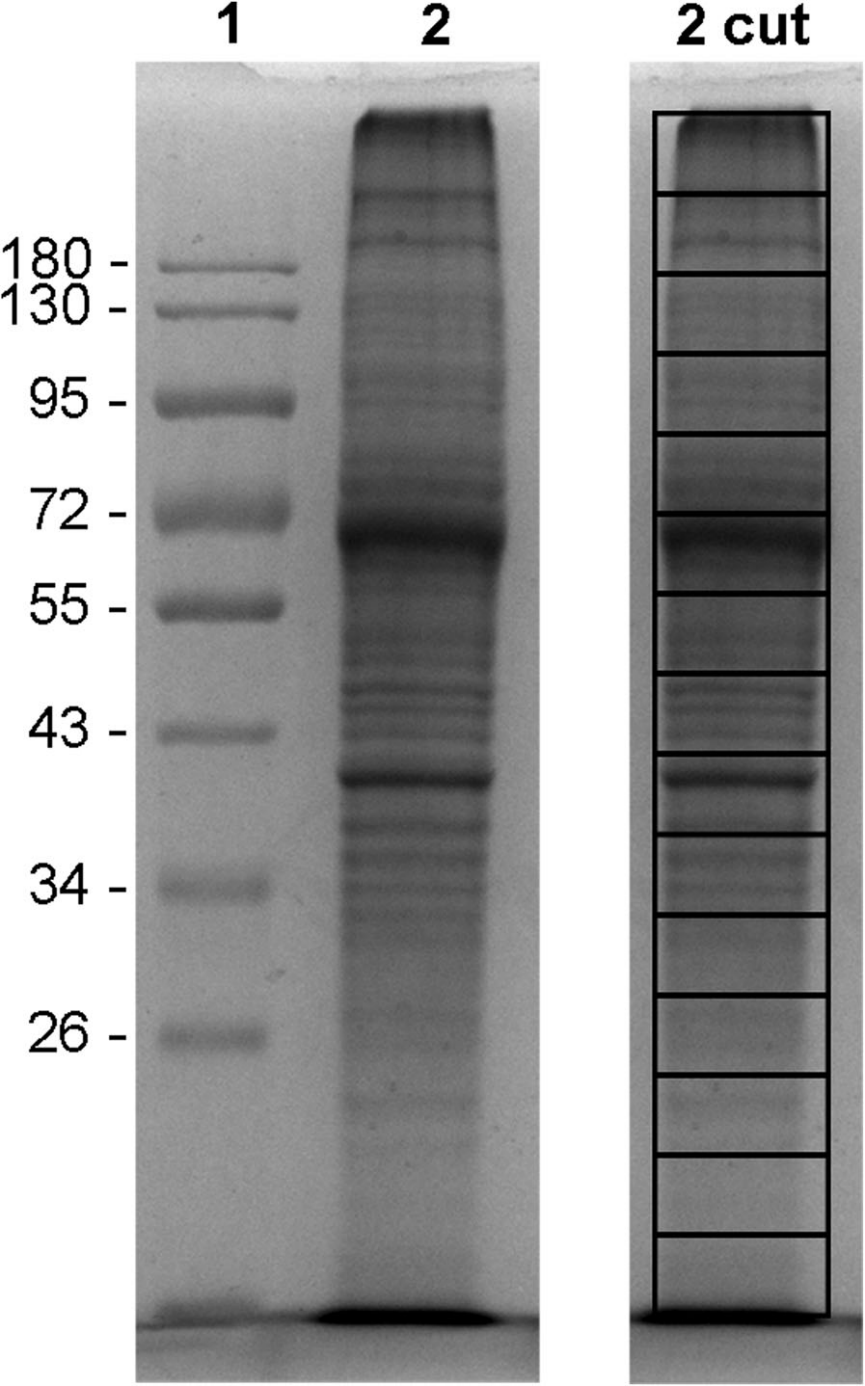
SDS-PAGE of the Lopburi virus protein lysate. The Lopburi02 virus cultured in Vero cells was concentrated by ultracentrifugation, lysed by lysis buffer containing SDS and resolved by SDS-PAGE (lane 2). The gel was cut along its length into 15 pieces for mass spectrometric analysis (lane 2 cut). PageRuler protein marker (Thermo Scientific) was used as a protein size marker (lane 1)

To further identify the unknown viruses, the Lopburi02 virus was propagated, and proteins were extracted from the concentrated virus. The whole protein lysate, composed of host and viral proteins (Fig. 5), was analysed via gel-based LC-MS/MS. After searching the NCBI protein database using the Mascot program focussing on viral proteins, it was suggested that Lopburi02 is an orthoreovirus (Table 2). The top five protein hits were matched to structural and non-structural proteins of PRV, Melaka, Pulau and Sikamat orthoreoviruses. After obtaining the LC-MS/MS result, primers (Additional file 1: Table S1) were designed on the basis of the S1 genomic sequence of the Melaka orthoreovirus. The primers were used in RT-PCR with RNA extracted from the culture supernatants of the Lopburi01 and Lopburi02, and results showed that both viruses are PRV.

**Table 2:** Top 5 of the Lopburi02 viral proteins analysed by LC-MS/MS and Mascot program

No.	Protein accession no.	Description	Organisms
1	gi|459014590	Major outer capsid (M2)	Melaka orthoreovirus
2	gi|38194450	Non-structural protein sigma NS (S3)	Pulau reovirus
3	gi|355477204	Sigma 2 (S4)	Orthoreovirus Sikamat/MYS
4	gi|38194452	Major outer capsid protein sigma 2 (S4)	Pulau reovirus
5	gi|459014574	Inner core shell (L3)	Melaka orthoreovirus

### Whole-genome analysis of the Lopburi orthoreoviruses

Sets of overlapping primers were designed on the basis of the genome sequences of the Melaka orthoreovirus (Additional file 1: Table S1) to amplify all 10 genome segments of the Lopburi01 and Lopburi02 viruses for nucleotide sequencing. After sequence analysis via nucleotide sequence alignment and contig assembly, all 10 whole-genome segment sequences of the Lopburi01 and Lopburi02 viruses were retrieved and submitted to the NCBI GenBank database. Table 3 shows the GenBank accession numbers and percent nucleotide and protein sequence identities of the two newly isolated viruses. Lopburi01 and Lopburi02 are identical with nucleotide and protein sequence identities of 99.7–100%. The nucleotide sequences of all segments were subjected to BLAST searches using the NCBI database. Published nucleotide sequences that exhibited the highest percent identities to sequences of the isolated virus are presented in Table 4. The identities with the reference sequences were 93.5–98.9% in all 10 segments. Re-assortments within the species group were observed in the Lopburi orthoreovirus genome. Segment L1 of the Lopburi virus is most identical to that of the bat-origin Pulau orthoreovirus of Malaysia. Segments L2, M3 and S3 are closely related to those of the bat-origin Cangyuan orthoreovirus of China. Segments L3 and M2 are related to those of the human-origin Melaka orthoreovirus of Malaysia. Segments M1 and S2 are closely related to those of the human-origin Kampar orthoreovirus of Malaysia. Segments S1 and S4 are closely related to those of the human-origin Sikamat orthoreovirus of Malaysia.

**Table 3:** Accession numbers of 2 newly identified orthoreovirus isolates and their % sequence identities in coding regions

	L1	L2	L3	M1	M2	M3	S1	S2	S3	S4
Lopburi01	KY751007	KY751009	KY751011	KY751013	KY751015	KY751017	KY751019	KY751021	KY751023	KY751025
Lopburi02	KY751008	KY751010	KY751012	KY751014	KY751016	KY751018	KY751020	KY751022	KY751024	KY751026
^a^% identity	99.9	100	99.8	99.9	99.7	100	100	100	100	99.8
^b^% identity	99.9	100	99.7	99.7	100	100	100	100	100	99.7

^a^% identity of nucleotide sequences

^b^% identity of translated protein sequences

**Table 4:** Highest nucleotide identities for each gene segment of the Lopburi02 orthoreovirus retrieved from the BLAST search

Segment	Encoded protein	% identity	Ref. strain	Host	Country	Year	Acc. no.
L1	λC or λ3	93.5	Pulau	Bat	Malaysia	1999	JF342666.1
		92.9	Cangyuan	Bat	China	2012	KM382259.1
L2	λB or λ2 (RNA pol)	98.2	Cangyuan	Bat	China	2012	KM382260.1
L3	λA or λ1	97.9	Melaka	Human	Malaysia	2006	JF342662.1
M1	μA or μ1	96.2	Kampar	Human	Malaysia	2006	JF342657.1
		95.8	Melaka	Human	Malaysia	2006	JF342663.1
M2	μB or μ2	95.8	Melaka	Human	Malaysia	2006	JF342664.1
M3	μNS	98.9	Cangyuan	Bat	China	2012	KM382264.1
S1	p10, p17 and σC	95.5	Sikamat	Human	Malaysia	2010	JF811580.1
S2	σA or σ1	98.7	Kampar	Human	Malaysia	2006	EU448335.1
S3	σNS	97.7	Cangyuan	Bat	China	2012	KM382267.1
		97.7	Xi river	Bat	China	2010	GU188275.1
S4	σB or σ2	97.2	Sikamat	Human	Malaysia	2010	JF811583.1
		96.7	Cangyuan	Bat	China	2012	KM382268.1

*L* large segment, *M* medium segment, *S* small segment

**Fig. 6:**
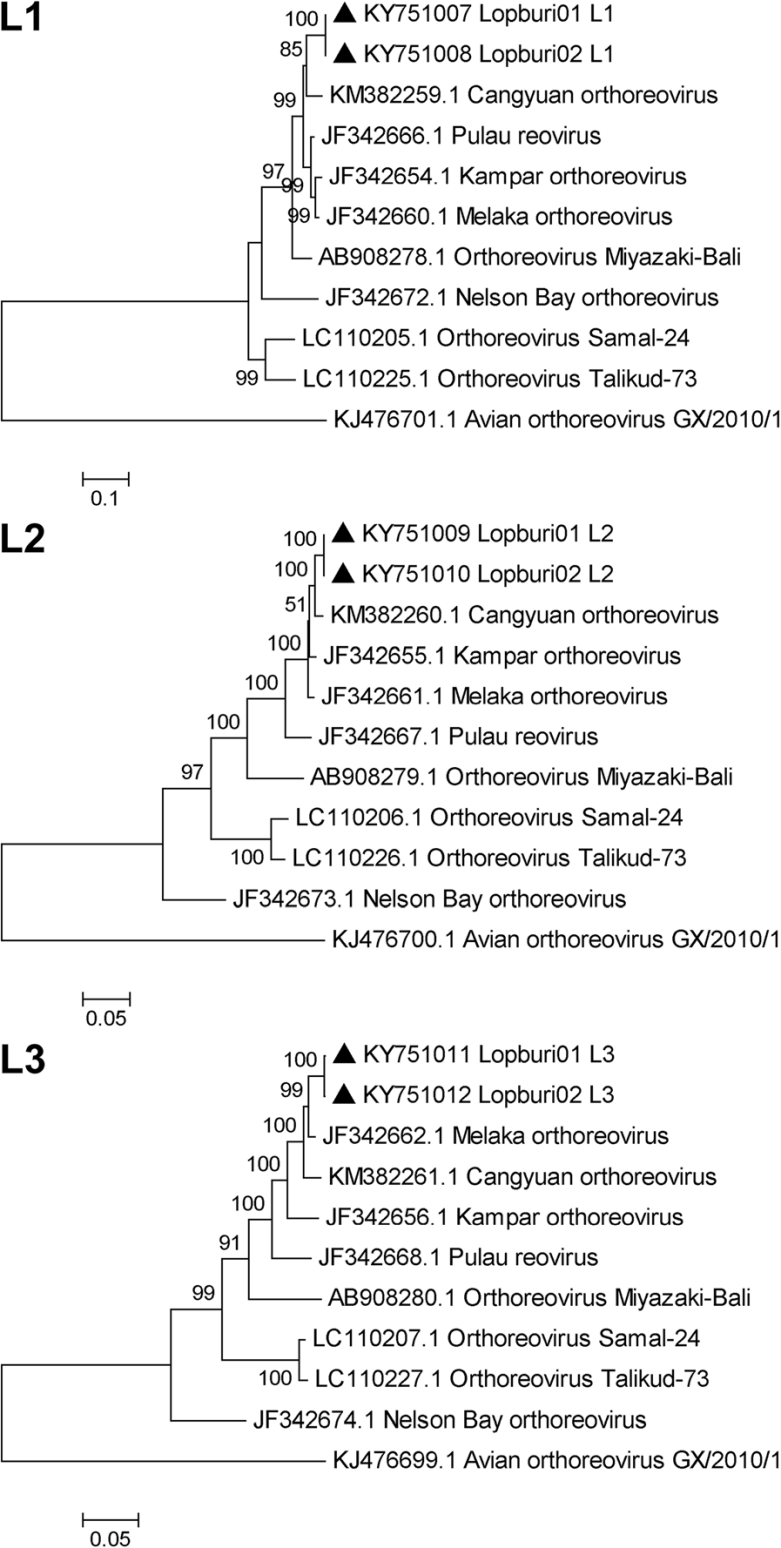
Phylogenetic trees based on nucleotide sequences of the whole L segments (L1–L3) of pteropine orthoreovirus. Phylogenetic trees were constructed using the maximum likelihood method and 1000 bootstrap replicates. Virus names and nucleotide sequence accession numbers obtained in this study are marked with triangles. The scale bar represents the number of nucleotide substitutions per site. Bootstrap values greater than 50 are indicated at the nodes. Sequences of the avian orthoreovirus GX/2010/1 were used as outgroups

**Fig. 7:**
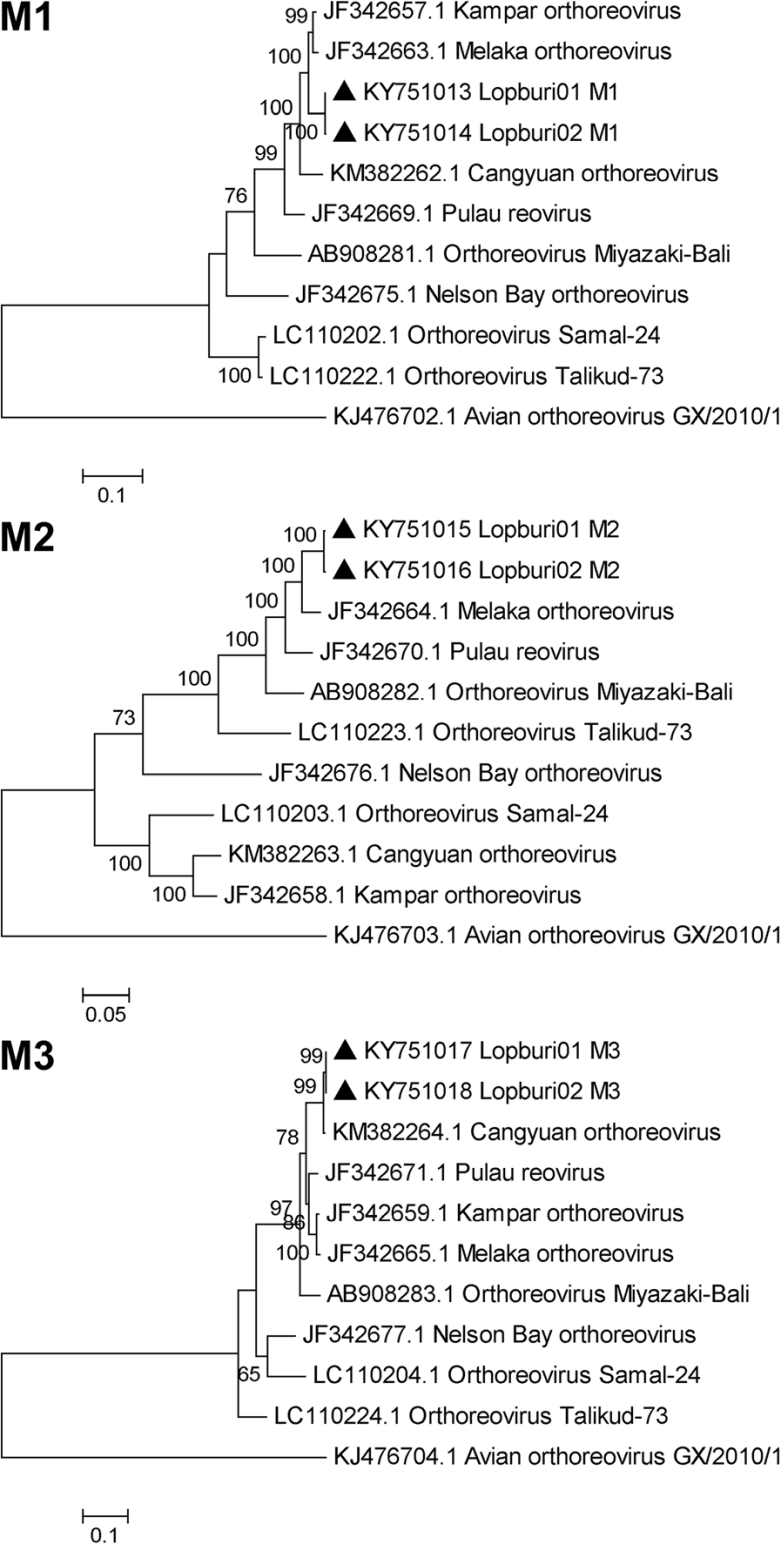
Phylogenetic trees based on nucleotide sequences of the whole M segments (M1–M3) of pteropine orthoreovirus. Phylogenetic trees were constructed using the maximum likelihood method and 1000 bootstrap replicates. Virus names and nucleotide sequence accession numbers obtained in this study are marked with triangles. The scale bar represents the number of nucleotide substitutions per site. Bootstrap values greater than 50 are indicated at the nodes. Sequences of the avian orthoreovirus GX/2010/1 were used as outgroups

**Fig. 8:**
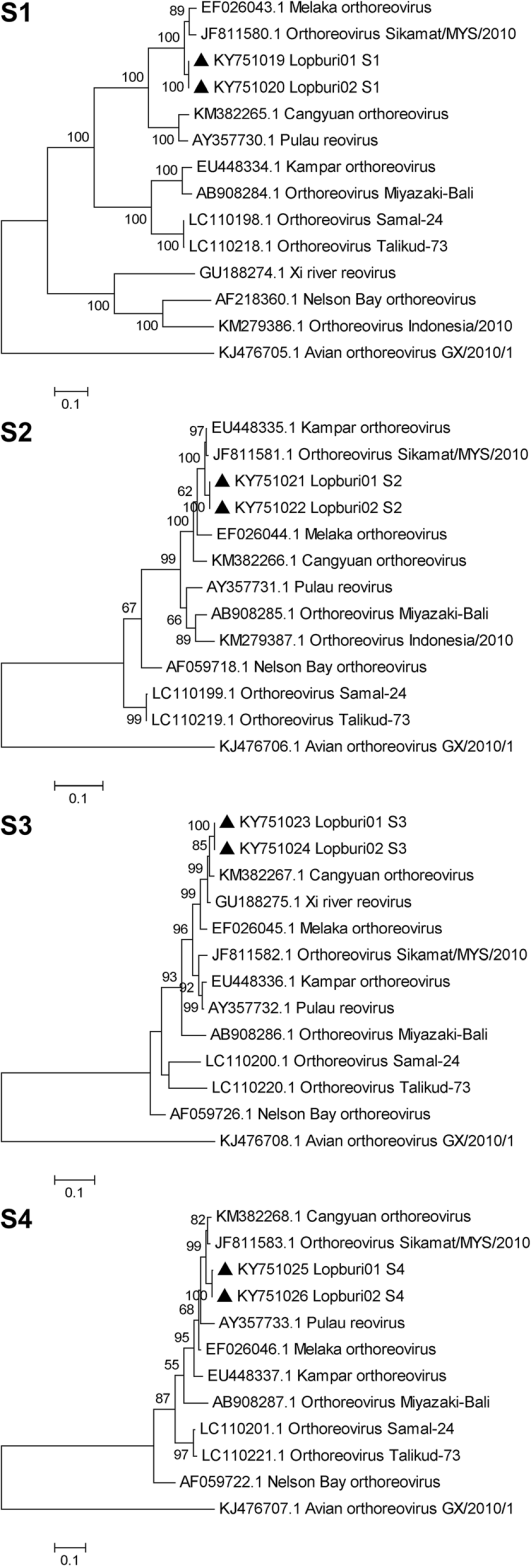
Phylogenetic trees based on nucleotide sequences of the whole S segments (S1–S4) of pteropine orthoreovirus. Phylogenetic trees were constructed using the maximum likelihood method and 1000 bootstrap replicates. Virus names and nucleotide sequence accession numbers obtained in this study are marked with triangles. The scale bar represents the number of nucleotide substitutions per site. Bootstrap values greater than 50 are indicated at the nodes. Sequences of the avian orthoreovirus GX/2010/1 were used as outgroups

Phylogenetic trees constructed using the maximum likelihood method are shown in Fig. 6 (L1, L2 and L3), Fig. 7 (M1, M2 and M3) and Fig. 8 (S1, S2, S3 and S4). The completed nucleotide sequences of the Lopburi01 and Lopburi02 viruses were compared with those of other PRV retrieved from the public database. Phylogenetic tree analysis demonstrated that the Lopburi orthoreoviruses are closely related to the Cangyuan, Melaka, Kampar and Sikamat orthoreoviruses that were identified in 2006–2012 in China and Malaysia. The newly identified Lopburi viruses most strongly resemble the Cangyuan virus with five segments (L1, L2, M3, S3 and S4) clustered together. The viruses are relatively separated from the prototype Nelson Bay orthoreovirus and the recently described Samal and Talikud PRVs from the Philippines.

Considering the nucleotide and protein sequence identities among the PRV sequences subjected to analysis, Table 5 presents the percent identities for each genome segment. The most conserved genome segment was S2 encoding a major inner capsid protein (96.3–100% amino acid identity). The most variable genome segment was S1, which contains three coding regions, p10 (membrane fusion protein), p17 and sigma C (cell attachment protein). The nucleotide sequence identities for these regions were 65.9–100%, 54.7–100% and 47.6–100%, respectively, and the amino acid sequence identities were 69.4–100%, 44.5–100% and 36.4–100%, respectively. Table 5 also presents percent sequence identities of each genome segment of the Lopburi viruses compared with those of the 5 closely related orthoreoviruses Pulau, Cangyuan, Melaka, Kampar and Sikamat orthoreoviruses.

**Table 5:** Ranges of % nucleotide and translated protein sequence identities compared between PRV sequences used for the phylogenetic analysis and between Lopburi viruses and 5 most closely related strains

Segment	% nucleotide identity						% amino acid identity					
	All strains	Pulau	Kampar	Melaka	Sikamat	Cangyuan	All strains	Pulau	Kampar	Melaka	Sikamat	Cangyuan
L1	80.6–99.9	93.4–93.5	92.3	92.7	–	92.9	91.1–99.9	97.7–97.8	97.5	97.1–97.2	–	97.7–97.8
L2	83.2–100	94	97.8	97.7	–	98.2	95.5–100	98.6	99.2	99.2	–	99.6
L3	84.0–99.8	92.6–92.7	94.2–94.3	97.9	–	96.7	96.1–99.7	98.4–98.5	98.8–98.9	99.3	–	99.3
M1	81.5–99.9	91–91.1	96.1–96.2	95.8	–	92.6–92.7	92.0–99.7	96.5–96.8	98.3–98.6	98.6–98.9	–	97.8–98
M2	77.6–99.7	93.3–93.4	78.1–78.2	95.6–95.7	–	78.5	93.6–100	99.7	94.9	100	–	95.1
M3	81.1–100	93.5	93.4	93	–	98.9	90.3–100	98.2	98.4	97.9	–	99.3
S1 p10	65.9–100	88.8	82.6	97.5	96.5	87.1	69.4–100	98.9	93.6	100	98.9	98.9
S1 p17	54.7–100	86.9	75.5	97.4	97.4	87.8	44.5–100	91.6	82.5	96.5	96.5	90.9
S1 σC	47.6–100	76.4	57.3	94.6	93.8	76.3	36.4–100	78.4	55.4	95.4	93.9	79
S2	82.8–100	91.4	98.6	94.7	98.3	94.6	96.3–100	98.3	99.7	99.2	99.5	98.5
S3	84.1–100	93	92.3	95.4	92.3	97.6	93.7–100	98.9	98.6	99.7	99.1	98.9
S4	81.3–99.8	92.1–92.3	91.9	95–95.2	96.8–97	96.3–96.5	88.6–99.7	92.7–93	96.3–96.6	98–98.3	98.8–99.1	99.1–99.4

### Detection of PRV and HEV RNA in monkey faecal samples and monkey species identification

Semi-nested RT-PCR was used to detect PRV and HEV RNA in the 55 faecal samples. Six samples (10.9%) were PRV positive according to the RT-PCR targeting PRV S1 segment. Fourteen samples (25.5%) were HEV positive according to the semi-nested RT-PCR targeting HEV ORF1 (Table 1). HEV-positive samples were detected from all five sampling sites, whereas PRV-positive samples were detected from four sites. Sequencing and BLAST analysis of the nested PCR products confirmed the detection of PRV and HEV sequences from monkey faeces.

PCR of mitochondrial 12S rRNA gene was used to confirm the monkey species in all PRV- and HEV-positive samples. The results of nucleotide sequencing and BLAST search revealed that the monkey species of all PRV- and HEV-positive samples is *Macaca fascicularis*.

## Discussion

In Lopburi Province, Thailand, macaques live in close proximity to human communities, including temples, schools, houses and working areas. Despite efforts to avoid close contact with animals, the potential for contact with animal faeces excreted into the environment remains. Four species of macaques reside in Lopburi Province, long-tailed or cynomolgus macaques (*Macaca fascicularis*), rhesus macaques (*Macaca mulatta*), pig-tailed macaques (*Macaca nemestrina*) and stump-tailed macaques (*Macaca arctoides*), with long-tailed macaques being a major species. In this study, two unknown viruses were isolated from monkey faeces. The isolates demonstrated a syncytial CPE on A549 and Vero cells. Primers specific for viruses possibly found in monkey fluids and faeces such as herpesviruses and enteric viruses, including enterovirus, norovirus, rotavirus, enteric adenovirus and astrovirus, were used in PCR and RT-PCR for the isolated viruses, but negative results were obtained. Subsequently, mass spectrometry was applied, and the viruses were successfully identified as PRVs named Lopburi01 and Lopburi02. Previously, 2D gel electrophoresis for protein separation and HPLC-MS/MS was used to identify an unknown virus from a plant extract [21]. In this study, we demonstrated that a simple SDS-PAGE for protein size separation followed by LC-MS/MS of trypsin-digested peptides and protein database searching can also be used to identify unknown culturable viruses. Furthermore, on performing RT-PCR for all monkey samples available, PRV was detected in 10.9% (6 of 55) of the faecal samples collected from different sites in the Mueng District of Lopburi Province. The analysis of the mitochondrial 12S rRNA gene in the monkey stool DNA confirmed that the host species from which the Lopburi orthoreoviruses were isolated was *M. fascicularis*.

The identification of the Lopburi orthoreoviruses by MS was in agreement with the physicochemical properties and morphological characteristic of the viruses observed by other assays. The isolates were identified as non-enveloped viruses that exert syncytial CPEs on A549 and Vero cells, which is a characteristic of PRVs [3]. The viruses are resistant to chloroform, which is characteristic of non-enveloped viruses, and acidic conditions, facilitating their persistence in faeces [4]. Incubation at pH 3 and pH 5 promoted the growth of the virus, as indicated by increases of log_10_ TCID_50_ compared with that observed in the control.

This study marks the first detection of an orthoreovirus in monkey faeces from Thailand. Phylogenetic analysis of the isolates indicated that Lopburi01 and Lopburi02 are the same virus with sequence identities of ≥99.7% in all genome segments. Of the 10 segments of orthoreovirus genomes, all but S1 contain one ORF encoding a structural or non-structural protein. S1 is polycistronic, as it can encode 1–3 proteins depending on the orthoreovirus species [3]. S1 of PRV is tricistronic with three overlapping ORFs, and it encodes p10 (membrane fusion protein), p17 and sigma C (cell attachment protein). Among these segments, S1 exhibits the greatest variability [14, 16]. In our analysis, the nucleotide and amino acid sequence identities were 47.6% and 36.4%, respectively. Furthermore, considering differences in the topology patterns of the phylogenetic trees, it can be suggested that genetic re-assortment occurred among orthoreovirus species. The Lopburi orthoreoviruses contain genome sequences related to those of the Pulau (L1), Kampar (M1 and S2), Melaka (L3 and M2) and Sikamat (S1 and S4) viruses of Malaysia and the Cangyuan virus (L2, M3 and S3) of South China with sequence identities of 93.5–98.9%. It can be speculated that a geographical hindrance may have played a role in the re-assortment, as the orthoreovirus from Thailand is more similar to viruses from Malaysia and China than to those from the Philippines (Samal and Talikud viruses), which is separated from Thailand by the Pacific Ocean.

As the Lopburi viruses were isolated from monkey faeces collected from the ground, the impact of the viruses on the animals’ health could not be demonstrated. Their role in human diseases also requires further investigation. Recently, a report illustrated the detection of PRV nucleic acids in 17% of oropharyngeal swabs collected from outpatients in Malaysia with acute upper respiratory tract infection. The viral agents were related to the Melaka and Kampar orthoreoviruses [28]. A sero-survey in Central Vietnam demonstrated that 4.4% of serum samples were PRV IgG positive [29]. These data were important because Vietnam shares a border with China. These findings emphasise that PRV is a bat-borne zoonotic virus that has high potential to cause cross-species infection in humans.

Another important issue is the possibility of monkey faeces contamination by PRV-positive bat excreta before the time of sample collection. Thailand is home to 139 different bat species, including fruit bats and flying foxes of the genera *Pteropus*, *Rousettus* and *Eonycteris* [30, 31], from which PRVs were isolated [6, 7, 13–16]. Fruit bats visit human areas to feed on fruit trees; in this case, the areas occupied by humans, monkeys and bats may overlap. In addition to PRV, the bats were infected with other viruses such as coronaviruses and the Nipah virus [30, 31]. Therefore, even if the detection of PRV in monkey faeces was due to contamination by bat excreta, the possibility of monkey faeces as an indirect source of zoonotic diseases cannot be eliminated. However, by analysis of the mitochondrial 12S rRNA gene, bat DNA was not detected in the faecal samples in this study, thus ruling out the contamination of the monkey samples with bat faeces.

Besides the detection of PRV by virus culture and RT-PCR, this particular set of monkey samples was originally intended for HEV detection. It was reported that NHPs such as macaques and chimpanzees can be experimentally infected by four serotypes of HEV, serotypes 1 and 2 found in humans and serotypes 3 and 4 found in humans and swine [32, 33]. A productive infection with seroconversion and viral shedding in faeces was demonstrated in cynomolgus macaques without clinical symptoms, indicating that the infected NHPs can serve as asymptomatic carriers of HEV [27]. In total, 25% of the faecal samples collected in this study were HEV RNA-positive, suggesting that monkeys in the wild could be reservoirs for HEV. Unfortunately, the HEV genotypes could not be identified because the short RT-PCR product (85 bp) was not suitable for genotype analysis. However, the detection of HEV RNA with a high detection rate in monkey faeces, even higher than that reported in pigs, the primary reservoir [34–36], can be used as a preliminary information that warrants a further investigation to conclude that monkey faeces are a source of HEV.

## Conclusions

We have detected and isolated PRV in *M. fascicularis* faeces collected from human areas in Thailand. The usefulness of the gel-based LC-MS/MS technique for identifying unknown culturable viruses was demonstrated. Whole-genome characterisation of the new PRV, the Lopburi orthoreovirus, was described. In addition, HEV RNA was detected in monkey faeces. Our results emphasise monkeys, especially a long-tailed macaque, as potential reservoirs of zoonotic viruses, such as PRV and possibly HEV, and suggest the need for systemic surveys of zoonotic viruses, especially in areas where human and animal habitats overlap.

## Methods

### Faecal samples

Fifty-five monkey faecal samples were collected in 2013 from the ground in five sites in Mueng District, Lopburi Province, Thailand (Fig. 1 and Table 1). The identities of the monkey faeces were provisionally confirmed by veterinarians according to their appearance. Monkeys found in the sample collection areas were macaques (*Macaca* spp.). No contact with the animals occurred during the collection process. The samples were transferred on ice and stored at − 80 °C until further processing. Faeces were processed by adding phosphate-buffered saline (PBS, pH 7.2) to produce 30% (*w*/*v*) solutions, which were mixed by vortexing and sonicated for 10 min at 4 °C before centrifugation at 1000×*g* for 15 min at 4 °C. Supernatants were collected and stored at − 80 °C.

### Virus isolation in A549 cell

Virus isolation was performed in the human lung epithelial cell line A549 (ATCC CCL-185). Supernatants of the 30% faecal suspension (w/v) in PBS were filtered through 0.45-μm syringe filters after debris removal by centrifugation at 1000×*g* for 15 min at 4 °C. One hundred microlitres of the filtrates were added to the A549 cell monolayers (80–90% confluent) grown in a 24-well tissue culture plate and incubated at 37 °C in a 5% CO_2_ atmosphere for 1 h. The inocula were discarded, and the cells were washed with PBS prior to the addition of minimum essential medium (MEM) supplemented with 2% foetal bovine serum (FBS), penicillin-streptomycin solution and insulin-transferrin-selenium-ethanolamine (ITS-X) (Gibco, Thermo Fisher Scientific), the latter of which was added according to a method for HEV isolation [37]. The cells were incubated at 37 °C in a 5% CO_2_ atmosphere and observed daily for 5 days for the presence of CPE and compared with the observations in control cells. Two sub-passages were performed before the cultures were discarded. Supernatants from CPE-positive samples were collected and stored at − 80 °C for further analysis.

### Virus propagation in Vero cell

Virus propagation was performed in African green monkey kidney cells (Vero) (ATCC CCL-81), which are regularly used in our laboratory. The CPE-positive culture supernatants were diluted (1:10) in Dulbecco’s modified Eagle’s medium (DMEM) supplemented with 2% FBS and penicillin-streptomycin solution (2% FBS DMEM). Then, 1.5 mL of the diluted supernatants was added to the Vero cell monolayers in T75 tissue culture flasks. After 1 h, the inocula were discarded, and the cells were washed with PBS. Next, 2% FBS DMEM was added, and the cells were incubated at 37 °C in a 5% CO_2_ atmosphere. Supernatants containing viruses were collected when a CPE of 80–90% was observed. The culture supernatants were stored at − 80 °C.

### Growth kinetics of Lopburi viruses in A549 and Vero cells

A549 and Vero cells were infected with Lopburi01 and Lopburi02 viruses at MOI of 0.5. Virus culture supernatants were collected at 0, 6, 12, and 24 h after the virus adsorption step and subjected to virus titration in Vero cells.

### Viral resistance to temperature, chloroform and acid

Viral resistance to temperature, chloroform and acid was determined via end-point viral titration in Vero cells. For temperature treatment, the viral culture supernatants were incubated at 50 °C, 60 °C or 70 °C for 1 h prior to viral titration. The supernatant stored at 4 °C was used as a control. For chloroform treatment, the viral culture supernatants were mixed with chloroform at a ratio of 1:1 for 30 or 60 min. The mixtures were subsequently centrifuged at 600×*g* for 5 min, and the upper aqueous phases containing viruses were collected for viral titration. The untreated supernatant was used as a control. For pH treatment, the viral culture supernatants were mixed with McIlvaine’s phosphate/citrate buffer pH 3 or pH 5 at a ratio of 1:10 [38] and incubated for 20 h at room temperature. Supernatant mixed with PBS (pH 7.2) was used as a control. Viral resistance to heat, chloroform and acid was determined by comparing the TCID_50_ between treated and untreated samples.

### Viral titration

The virus culture supernatants were serially diluted 10-fold in 2% FBS DMEM. Fifty microlitres of the diluted viruses were added to Vero cell monolayers cultured in a 96-well plate (total volume, 200 μL). The cultures were incubated at 37 °C in a 5% CO_2_ atmosphere. The CPE was assessed daily until day 3 as an end-point compared with that in control wells. The titration was performed in quadruplicate wells for each viral dilution. TCID_50_/mL was calculated using the Spearman and Kärber algorithm [39].

### Electron microscopy

Vero cells infected with the Lopburi02 virus were collected by centrifugation at 1000×*g* for 15 min at 4 °C. Primary and secondary fixations of the pellet were achieved via a 1-h exposure to 2.5% glutaraldehyde and 1% osmium tetroxide in 0.1 M sucrose phosphate buffer, respectively. After washing, the pellet was dehydrated via a graded ethanol series, infiltrated in LR White resin (EMS®), embedded in capsule beams and finally polymerised at 65 °C for 48 h. All embedded cells were sectioned at 100-nm thickness and stained with uranyl acetate and lead citrate. The specimen was examined under a transmission electron microscope (HT7700; Hitachi, Japan).

### Virus concentration by ultracentrifugation for protein preparation

Fifty millilitres of the virus supernatant cultured in Vero cells were collected, and cell debris was removed by centrifugation at 1000×*g* for 15 min at 4 °C. Viruses in the supernatant were concentrated by ultracentrifugation in polycarbonate centrifuge bottles (no. 355603, Beckman Coulter) using a Beckman L7–65 ultracentrifuge (rotor 70.1 Ti) set at 35,000 rpm for 1.5 h at 4 °C. After ultracentrifugation, pellets containing viruses were processed for mass spectrometric analysis via re-suspension in lysis buffer (1% NaCl, 1% SDS and 1% Triton-X) to produce a virus protein lysate.

### LC-MS/MS

The virus protein lysate was size-separated via 12% SDS-PAGE, and the gel was stained with the Coomassie brilliant blue G250 solution (Bio-Rad). The gel was cut along its length into 15 pieces (Fig. 5), and each piece was cut into equal small cubes and separately de-stained with a de-staining solution [50 mM NH_4_HCO_3_, 50% (*v*/v) acetonitrile (ACN)]. Gel cubes containing proteins were treated with 5 mM dithiothreitol (DTT) (GE Healthcare) and alkylated in 250 mM iodoacetamide (IAM) (GE Healthcare). The gels were then incubated for 30 min in the dark prior to dehydrating with 200 mL of ACN and digesting with trypsin (100 ng/mL) (Sigma-Aldrich). After tryptic digestion, peptides were extracted from the gels using 50% (v/v) ACN and dried using a vacuum evaporator. The peptides were resuspended in 0.1% formic acid and analysed using a MicroToF Q II mass spectrometer (Bruker). The front end of the mass spectrometer was coupled to an Ultimate 3000 nano-LC system (Dionex). After separation, peptide fractions were automatically infused into the mass spectrometer. LC-MS/MS raw data files were generated and converted into mascot generic format (.mgf) files using DataAnalysis™ software version 3.4. The .mgf files were searched using the Mascot program version 2.4.1 (Matrix Science) against the US National Center for Biotechnology Information (NCBI) database. The organism for searching was set as ‘virus’. The maximum number of missed cleavages was set to 1. Peptide tolerance and tandem MS tolerance were set to 1.2 and 0.6 Da, respectively. The fixed modification was set to cysteine carbamidomethylation, and variable modification included methionine oxidation. All reported peptides showed a more than 95% confidence level.

### Orthoreovirus RT-PCR for whole-genome sequencing and virus detection

For amplification of orthoreovirus genome segments for nucleotide sequencing, total RNA was extracted from 140 μL of viral culture supernatants using a QIAamp Viral RNA Mini Kit. RT-PCR using QIAGEN OneStep RT-PCR Kit was performed to amplify overlapping fragments of PCR products for each orthoreovirus genome segment. Primers for orthoreovirus whole-genome amplification were based on published sequences of the Melaka orthoreovirus and provided in Additional file 1: Table S1. RT-PCR was performed in mixtures containing 1× QIAGEN OneStep RT-PCR buffer, 0.4 mM dNTPs, 0.6 μM each of forward and reverse primers, 20 units of RNaseOUT™ Recombinant Ribonuclease Inhibitor (Invitrogen), 1 μL of QIAGEN OneStep RT-PCR Enzyme Mix and 3 μL of RNA in a total volume of 25 μL. The RT-PCR protocol was 50 °C for 30 min for the reverse transcription step followed by 95 °C for 15 min, 40 cycles of 95 °C for 30 s, 50 °C for 30 s and 72 °C for 45–90 s (depending on the product length; approximately 1 min/1 kb) and a final extension step of 72 °C for 10 min.

For orthoreovirus detection in monkey faecal samples, RNA extracted from 140 μL of the 30% faecal solutions was subjected for the first step RT-PCR with primers S1 Reo1 F and S1 Reo1 R (Additional file 1: Table S1) as mentioned above. A semi-nested PCR was performed with primers S1 Reo1 F and S1 Reo1 nested R (5′GCCTGACATATCCGCGRGTT3′) in a reaction containing 1× Standard PCR buffer, 0.2 mM dNTPs, 0.2 μM each of forward and reverse primers, 2.5 units of *Taq* DNA polymerase (New England BioLabs) and 2 μL of the first step RT-PCR product in a total volume of 25 μL. The reaction condition was 95 °C for 2 min followed by 30 cycles of 95 °C for 30 s, 50 °C for 30 s and 72 °C for 30 s and a final extension at 72 °C for 10 min. The RT-PCR and semi-nested PCR product sizes were 661 and 276 bp, respectively. PCR products were resolved by gel electrophoresis in 1.5% agarose gels and observed under a UV transilluminator.

### Hepatitis E semi-nested RT-PCR

RT-PCR for detection of HEV in monkey faecal samples was performed with primers specific for the open-reading frame (ORF) 1 of HEV [37]. The RT-PCR reactions contained 1× OneStep RT-PCR buffer, 0.4 mM dNTPs, 0.6 μM each of forward (HE61; 5′-CACRTATGTGGTCGAYGCCATGGAG-3′) and reverse primers (HE51; 5′-GCCKRACYACCACAGCATTCG3–3′), 20 units of RNaseOUT™ Recombinant Ribonuclease Inhibitor (Invitrogen, Thermo Fisher Scientific), 1 μL of QIAGEN OneStep RT-PCR Enzyme Mix and 10 μL of RNA in a total volume of 25 μL. The reaction protocol was 50 °C for 30 min for reverse transcription followed by 95 °C for 15 min, 45 cycles of 95 °C for 15 s, 55 °C for 30 s and 72 °C for 30 s and a final extension at 72 °C for 10 min. Products from the first-round RT-PCR were subjected to semi-nested PCR in reactions containing 1× Standard PCR buffer, 0.2 mM dNTPs, 0.2 μM each of forward (HE50; 5′-AAGGCTCCTGGCRTYACWAC-3′) and reverse primers (HE51), 2.5 units of *Taq* DNA polymerase (New England BioLabs) and 0.5 μL of the first step RT-PCR product in a total volume of 25 μL. The reaction condition was 95 °C for 2 min followed by 20 cycles of 94 °C for 15 s, 60 °C for 30 s and 72 °C for 15 s and a final extension at 72 °C for 10 min. The expected product sizes of the first step RT-PCR and semi-nested PCR were 125 and 85 bp, respectively. The products were resolved by gel electrophoresis in 2% agarose gels and observed under a UV transilluminator. RNA from HEV-positive human faeces was used as a positive control. Nucleotide sequencing was performed using the nested PCR products to confirm amplification of the HEV sequence.

### PCR detection of other viruses

PCR for herpesvirus detection [40] and RT-PCR for enterovirus detection [41] were performed as previously described. Rotavirus, enteric adenovirus, norovirus and astrovirus were detected using a multiplex RT-PCR kit (Seeplex® Diarrhea-V ACE detection, Seegene) according to the manufacturer’s instructions.

### Nucleotide sequencing and phylogenetic analysis

RT-PCR product bands of expected size were excised from agarose gels and purified using a PureLink® Quick Gel Extraction Kit (Invitrogen). The purified products were submitted for direct nucleotide sequencing in two directions using forward and reverse primers by the Bioneer Corporation (Republic of Korea). To obtain nucleotide sequences at the 5′ and 3′ ends of the virus genome segments, PCR fragments were cloned into a pGEM®-T Easy vector (Promega) and sequenced from universal primer sites inside the vector. Nucleotide sequences were processed using BioEdit version 7.0.4.1. Sequence contigs derived from the two-directional sequencing were joined using the Contig Assembly Program. Basic Local Alignment Search Tool (BLAST) searches were used to determine the identities of the nucleotide sequences in comparison with those deposited in the NCBI GenBank nucleotide database. The nucleotide sequences were aligned using ClustalW. Phylogenetic trees were constructed in the MEGA 5 program. A maximum likelihood method based on Tamura-Nei model with 1000 bootstrap replicates was applied. The percent sequence identity was determined using the Sequence Identity Matrix function in BioEdit.

### Monkey species identification

DNA was extracted from monkey faecal samples using QIAamp Stool DNA Mini kit according to manufacturer’s recommendations. A mitochondrial 12S rRNA gene sequence of approximately 400 bp was amplified using PCR with primers 12S-L1091 5′-AAAAAGCTTCAAACTGGGATTAGATACCCCACTAT-3′ and 12S-H1478 5′-TGACTGCAGAGGGTGACGGGCGGTGTGT-3′ [42]. After visualisation on a 1.5% agarose gel, amplicons were subjected for nucleotide sequencing and the host species was analysed by BLAST searching of the nucleotide sequences with NCBI database.

## Additional file

Additional file 1: **Table S1.** Primers for the orthoreovirus whole-genome sequencing, designed based on sequences of the Melaka orthoreovirus. Sequences of primers for the orthoreovirus whole-genome sequencing. (DOC 85 kb)

## Abbreviations

2D: Two-dimensional
ACN: Acetonitrile
ARV: Avian orthoreovirus
BLAST: Basic Local Alignment Search Tool
CPE: Cytopathic effect
DMEM: Dulbecco’s modified Eagle’s medium
DTT: Dithiothreitol
FBS: Foetal bovine serum
HEV: Hepatitis E virus
HPLC-MS/MS: High-performance liquid chromatography-tandem mass spectrometry
IAM: Iodoacetamide
ITS-X: Insulin-transferrin-selenium-ethanolamine
LC: Liquid chromatography
LC-MS/MS: Liquid chromatography-tandem mass spectrometry
MEM: Minimum essential medium
MRV: Mammalian orthoreovirus
MS: Mass spectrometry
MS/MS: Tandem MS
NBV: Nelson Bay orthoreovirus
NCBI: National Center for Biotechnology Information
NHPs: Non-human primates
ORF: Open-reading frame
PRV: Pteropine orthoreovirus
TOF MS: Time-of-flight mass spectrometry
