# Supplementary material for: Mass spectrometry-based identification and whole-genome characterisation of the first pteropine orthoreovirus isolated from monkey faeces in Thailand
Source: BMC Microbiol. 2018 Oct 17;18:135. doi: 10.1186/s12866-018-1302-9 (PMC6192116; doi:10.1186/s12866-018-1302-9)
Supplement: Supplementary file 1 — Table S1. Primers for the orthoreovirus whole-genome sequencing, designed based on sequences of the Melaka orthoreovirus. Sequences of primers for the orthoreovirus whole-genome sequencing. (DOC 85 kb) [file 12866_2018_1302_MOESM1_ESM.doc]

**Table S1** Primers for the orthoreovirus whole genome sequencing, designed based on sequences of the Melaka orthoreovirus

| **Segment** | **Primer** | **Sequence (5’-3’)** | **Position** | **Product size (bp)** | **Ref. sequence** |
| --- | --- | --- | --- | --- | --- |
| S1 | S1 Reo1 F | TGTTCTCGAGTGTCGCTATGAG | 13-673 | 661 | EF026043.1 |
|  | S1 Reo1 R | GCTTATGCTCTGGGTCATCGT |  |  |  |
|  | S1 Reo2 F | CCCAGCAGCGAAGAGAAGTT | 618-1603 | 986 |  |
|  | S1 Reo2 R | TGAATAGCTGTYCTCGACGG |  |  |  |
| S2 | S2 Reo F | GCTTTAAGAACCACGATGGC | 1-1322 | 1322 | EF026044.1 |
|  | S2 Reo R | GATGATTAGACCACGGCTGAG |  |  |  |
| S3 | S3 Reo F | TCCTGTTGAGACGTGATCATGG | 11-1186 | 1176 | EF026045.1 |
|  | S3 Reo R | TAGCTCCTGTCGATGCTCAC |  |  |  |
| S4 | S4 Reo F | CTTATTTTGTCTTGGCGYGC | 2-1166 | 1165 | EF026046.1 |
|  | S4 Reo R | GCTAACCCTGTCCCAAGTGG |  |  |  |
| M1 | M1 Reo1 F | GCTTTAAATCGACATGGCGTACTT | 1-1206 | 1206 | JF342663.1 |
|  | M1 Reo1 R | CGACCATACTGCCAAGGCTT |  |  |  |
|  | M1 Reo2 F | TCACCTTGTTCTCGGATCGT | 1056-2250 | 1195 |  |
|  | M1 Reo2 R | CCACAGACGTCATAGCAGCA |  |  |  |
| M2 | M2 Reo1 F | GCTTATTTTGTGACGGATCGCA | 1-1074 | 1074 | JF342664.1 |
|  | M2 Reo1 R | AAGTTRTAGGCAACATCAAAACCCG |  |  |  |
|  | M2 Reo2 F | AGGTYTCTGAGGCRTTGTGG | 1008-2144 | 1136 |  |
|  | M2 Reo2 R | ATGAATAAGCGGACGGACCC |  |  |  |
| M3 | M3 Reo1 F | TGCTTCGCCATGTCACTAAG | 20-1004 | 985 | JF342665.1 |
|  | M3 Reo1 R | CAAATCCTAAGCACACACGAATG |  |  |  |
|  | M3 Reo2 F | ACCAGAGAAATGGCGACAAC | 849-1956 | 1108 |  |
|  | M3 Reo2 R | CTTTCCGTCGTGCTTCAAATC |  |  |  |
| L1 | L1 Reo1 F | TTTATCACTCATGGCTCAGATTCG | 3-1241 | 1239 | JF342660.1 |
|  | L1 Reo1 R | AGAGCTAGATCGACGGGAGA |  |  |  |
|  | L1 Reo2 F | CTCCCAAGCCTCGTTCCTTT | 1043-2107 | 1065 |  |
|  | L1 Reo2 R | GAGTCACTGAAGCTCCACGA |  |  |  |
|  | L1 Reo3 F | CAATTTCCCTACCTCACGGG | 1851-3054 | 1204 |  |
|  | L1 Reo3 R | CGGGAAGAATTGTGACAGCAC |  |  |  |
|  | L1 Reo4 F | AGACTTTTCCTGGTGCTACTGT | 2918-3871 | 954 |  |
|  | L1 Reo4 R | GGGTCAGCGTCTAGACTGGA |  |  |  |
| L2 | L2 Reo1 F | TGATCCTACCATGCATGTCAAAG | 8-1078 | 1070 | JF342661.1 |
|  | L2 Reo1 R | GTGTACGTCGCTGTYTTGGA |  |  |  |
|  | L2 Reo2 F | TGGCTTGAACCTCGTAGTGA | 909-2194 | 1286 |  |
|  | L2 Reo2 R | TTCCTGGTGGTTATCATGGAAC |  |  |  |
|  | L2 Reo3 F | GGATTCCGCGTAGTCAGTGT | 2131-3248 | 1118 |  |
|  | L2 Reo3 R | CGGATTGACTCCAGCACATAAC |  |  |  |
|  | L2 Reo4 F | GCTCAGTTACCTCGCAGTCC | 2844-3803 | 960 |  |
|  | L2 Reo4 R | CGACGCATTACTTGGCTGAT |  |  |  |
| L3 | L3 Reo1 F | GCTTTATTCCCCTAAGCGCG | 1-1222 | 1222 | JF342662.1 |
|  | L3 Reo1 R | CAGGGAGRACTCGCAACTG |  |  |  |
|  | L3 Reo2 F | CGTTCAACCCAAATACTTTCCGA | 1070-2251 | 1182 |  |
|  | L3 Reo2 R | GCACGTCTGGAGGAGTGAAA |  |  |  |
|  | L3 Reo3 F | CCGGCTAACATTGATCGTCG | 2107-3424 | 1318 |  |
|  | L3 Reo3 R | TCAAGCGAGGAAATATGCCGA |  |  |  |
|  | L3 Reo4 F | CTGTTGTGTTCACTCGTGGC | 3239-3946 | 708 |  |
|  | L3 Reo4 R | CTCCCGAGGGTGTTCTAGGA |  |  |  |
